# Association between pregnancy intention and optimal breastfeeding practices in the Philippines: a cross-sectional study^1^

**DOI:** 10.1186/1471-2393-12-69

**Published:** 2012-07-23

**Authors:** Valerie Gilbert T Ulep, Maridel P Borja

**Affiliations:** 1 Philippine Institute for Development Studies, Amorsolo St., Legaspi Village, Makati City, Philippines; 2 Department of Epidemiology and Biostatistics, College of Public Health, University of the Philippines, Pedro Gil St., Ermita, Manila, Philippines

## Abstract

**Background:**

The effect of pregnancy intention on post-natal practices like breastfeeding is still poorly understood in the Philippines. In this light, this study aims to determine the association between pregnancy intention and optimal breastfeeding practices in the Philippines.

**Methods:**

This is a cross-sectional study design using the 2003 Philippine National Demographic and Health Survey. Logistic regression analysis was used to determine the independent association of pregnancy intention and optimal breastfeeding practices. The study includes 3,044 last-born children aged 6–36 months at the time of survey. Dead children were also included as long as their age of death satisfies the age criterion.

**Results:**

Children born from mistimed pregnancies are more likely to have late breastfeeding initiation compared to children born from wanted pregnancies (OR = 1.44; 90%CI: 1.17-1.78). However, this occurs only among children belonging to households with low socio-economic status. Among children belonging to households with high socio-economic status, no significant effect of pregnancy intention on breastfeeding initiation was observed. Children born from unwanted pregnancies are less likely to have short breastfeeding duration (OR = 0.60; 90%CI: 0.48-0.76). However, this occurs only among children belonging to households with high socioeconomic status. No significant effect of pregnancy intention on breastfeeding duration was observed among children belonging to households with low socio-economic status.

**Conclusion:**

The findings of this study suggest that there are different effects of pregnancy intention on the two types of optimal breastfeeding practices examined. With regards to breastfeeding duration, it was found that among infants belonging to high SES, the odds of having short breastfeeding duration is lower among children born from unwanted pregnancies compared to children born from wanted one. Conversely, children belonging to low SES household, the odds of having late breastfeeding initiation among children born from mistimed pregnancies are higher compared to children born from wanted pregnancies.

## Background

The benefits of prolonged and early initiated breastfeeding are well documented. Prolonged and early breastfeeding have been proven to protect children against diseases, and play an important role in their cognitive development [1-3]. Despite the recognized health benefits, breastfeeding practices throughout the world remain suboptimal [4]. In the Philippines, only 64% of children are breastfed for more than six months and only 54% are breastfed within the first hour of life [5].

Maternal factors like pregnancy intention are important determinants of breastfeeding practices. Studies conducted in three different countries have demonstrated consistent results that unintended pregnancy is a risk factor of shorter breastfeeding duration. It is possible that women with unintended pregnancies may experience psychosocial stresses that inhibit them to practice desirable health behaviors [6-9]. On the other hand, no previous study was done to determine if pregnancy intention has an effect on first hour breastfeeding initiation.

In this study, we hypothesized that pregnancy intention may have different effect on optimal breastfeeding practices in the Philippines. We quantified the association between the pregnancy intention at the time of conception and optimal breastfeeding practices, both the first hour initiation and continuation to at least six months, for children 3 years old or younger at the time of survey.

## Methods

This study used the 2003 National Demographic and Health Survey, a cross sectional national survey implemented by National Statistics Office. Face to face questionnaire-guided interview was used to collect health and demographic related information from 13, 633 women of reproductive age. Three-stage sampling design was used to represent the country in seventeen regions. In the first stage, 819 villages in each region were selected using the 2000 Census master list. In the second stage, enumeration areas were selected in each village. An enumeration area is defined as an area with discernable boundaries and 150 neighboring households. In the third stage, an average of 17 households was selected from each enumeration area using systematic sampling. Actual data collection was carried out from June to September 2003 with a 99% response rate [5].

Secondary data that was formally requested from Macro International was used to answer the objectives of the study. Since no patient intervention was involved, there was no need for the study to undergo ethical approval. The respondents are identified through unique codes and neither by their names nor addresses to ensure confidentiality of respondent's identity.

Analysis was restricted to last-born children aged 6–36 month old at the time of survey. Children less than 6 months old (n = 639) were excluded since their breastfeeding duration cannot be determined, while children more than 36 months old (n = 1,237) were also omitted in the analysis to minimize possible recall bias. Dead children were included as long as their age of death satisfies the age criterion. Thus, out of 4,920 last-born children surveyed, only 3,044 children were eligible for inclusion.

Pregnancy intention was categorized into three categories: (1) wanted: mother wanted to become pregnant then, (2) mistimed pregnancy: mother want to wait later and (3) unwanted: mother did not want any more children. Mothers who responded “do not know” were excluded from the study. There were two breastfeeding outcomes in this study: breastfeeding duration and initiation. Mothers’ of children reported less than six months of breastfeeding were considered as having short breastfeeding duration, otherwise they were categorized as having prolonged duration of breastfeeding. On the other hand, mothers who answered less than one hour were considered as having initiated breastfeeding early otherwise were considered late.

Maternal socio-demographic, prenatal and delivery-related factors were considered as potential confounders. Maternal age at delivery was determined. Education was categorized as no education/primary, secondary and higher. Marital status was categorized as single (previously married) of currently married (including living-in together). Maternal employment was grouped into not working, working all year and working on a seasonal basis. Type of residence was categorized as either urban or rural. Type of delivery was categorized as normal vaginal or cesarean section. In this study, parity and socio-economic status were also assessed as possible effect measure modifiers. Parity was categorized as primaparity (only one live birth) and multiparity (2 or more live births). Socio-economic status was determined by principal component analysis. Ownership of seven household assets, namely, refrigerator, motorcycle, television, radio, electricity, automobile and radio were subjected to principal component analysis to derive socio-economic index. Mothers with zero or less socio-economic scores were considered poor otherwise categorized as non-poor [10].

**Figure 1:**
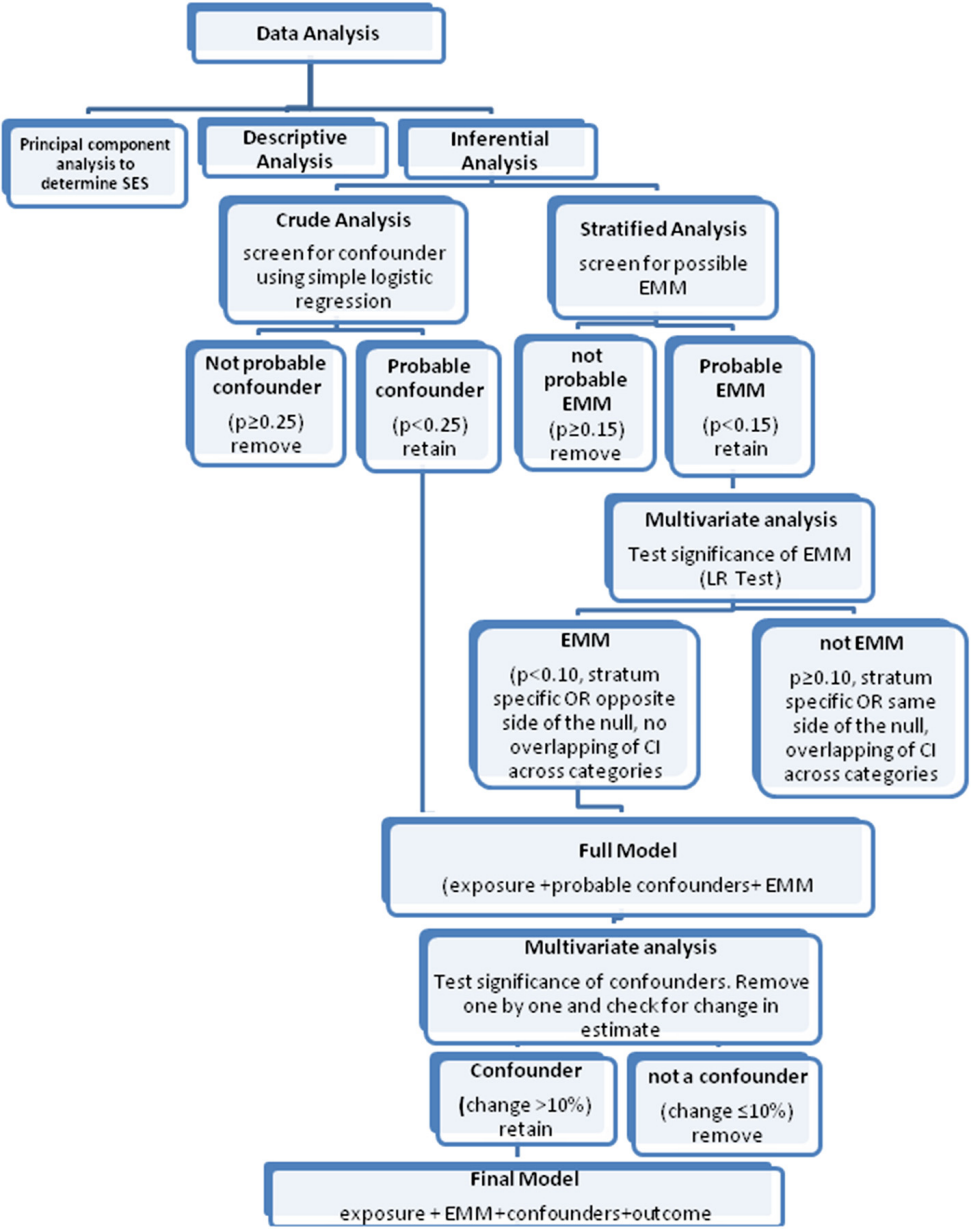
Algorithm of data analysis.

Figure 1 shows the different stages to determine the independent association between pregnancy intention and optimal breast feeding practices, namely, (1) bivariate analysis (2) stratified analysis and (3) multivariate analysis.

Bivariate analysis was used as a preliminary step in screening for the probable confounders. To be more conservative in the of possible confounders, those with a p-value less than 0.25 in at least one category were considered as probable confounders [11]. In the association between pregnancy intention and breast feeding initiation, place of residence, maternal education, employment, marital status, maternal age of conception and type of delivery variables were assessed as probable confounders. In the association between pregnancy intention and breastfeeding duration, same variables were considered as probable confounders in addition to breastfeeding initiation. Stratified analyses were also performed to determine if socio-economic status and parity were probable effect measure modifiers (EMM). A chi-square test of homogeneity with a p- value less than 0.15 qualifies a variable as a probable effect measure modifier [11].

Multivariate logistic regression analyses were performed to determine if EMM screened in stratified analysis and the probable confounders screened in the crude analysis were true effect measure modifiers and confounders. The significance of EMM screened in the stratified analysis were determined using Likelihood Ratio Test. Interaction terms were retained if the p-value generated was less than 0.10, indicating that the examined variable is indeed an effect measure modifier.

After assessment of effect measure modification, probable confounders identified during crude analysis were also assessed. The process of assessing confounders started with building the full model. The full contains the outcome variable (breastfeeding duration/initiation), the exposure variable (pregnancy intentions), all probable confounders and interaction terms. The probable confounder with the largest p-value was the first variable to be removed from the full model. In the presence of effect measure modifier as determined by the LR Test, stratum specific assessment of confounding was performed. Confounding was determined by comparing the odds ratio in the model with the deleted variables to that of the full model. If the odds ratio of the full model which contains the exposure, outcome and probable confounders differs from the OR of the reduced model by 10% or more in any of the stratum of the effect measure modifier, then the variable was considered a confounder.

## Results

In the total sample of 3,044, 1,482 (48.7%) were breastfed within the first hour of life while 2,118 (69.6%) were breastfed for at least six months. On the other hand, more than half of the children in the study were born from wanted pregnancies (52%). The others were born from either mistimed (24%) or unwanted pregnancies (24%).

Table 1 presents the results of bivariate analyses for identifying probable confounders on the association between pregnancy intention and breastfeeding practices. Mothers living in urban areas are more likely to initiate breastfeeding late compared to their counterparts living in rural areas. Similarly, mothers with high socio-economic status are also more likely to initiate their breastfeeding late than mothers with low socio-economic status. Maternal socio-demographic characteristics like educational attainment, employment and marital status were also assessed. Mothers with higher educational attainment are more likely to initiate breastfeeding late compared to mothers with secondary and primary education. Higher probabilities to initiate breastfeeding late were also observed among mothers working all year compared to their non-working counterparts and currently married compared to single mothers. For maternal-related factors, primiparous mothers are more likely to practice late initiated breastfeeding compared to their multiparous counterparts. Similarly, mothers who delivered via cesarean section are more likely to initiate their breastfeeding late. No difference on the average maternal age at delivery of mothers who initiated breastfeeding early compared with their counterparts who initiated breastfeeding late. All variables were considered as possible confounder except for maternal age at delivery since the p value is more than 0.25.

**Table 1:** Bivariate analysis of correlates as probable confounders on the association between pregnancy intention and optimal breastfeeding practices

**Correlates**	**Late initiation** **(more than 1 h)** **n = 1,482**		**Short Breastfeeding ** **(more than 6 months) ** **n = 926**	
	**Frequency (%)**	**OR**	**Frequency (%)**	**OR**
**Type of residence**				
Urban	767 (53.9%)	1.22	586 (41.2%)	2.64
Rural	795(49.0%)	Ref	340 (21.0%)	Ref
**Socio-economic status**				
High	823(54.5%)	1.29	651 (43.1%)	3.46
Low	739(48.2%)	Ref	275 (17.9%)	Ref
**Maternal educational attainment**				
Higher	458(56.0%)	1.37	404 (49.4%)	4.79
Secondary	643(50.6%)	1.11	360 (28.4%)	1.94
No formal education/primary	461(48.2%)	Ref	162 (17.0%)	Ref
**Maternal employment**				
working all-year	410 (49.8%)	1.01*	334 (40.9%)	1.91
working seasonal occasional	314 (57.8%)	1.36	145 (26.6%)	1.00*
not working	838 (50.2%)	Ref	447 (26.6%)	Ref
**Current marital status**				
Single/widowed/not living together	72 (58.1%)	1.33	55 (44.4%)	1.88
Married	1,490 (51.0%)	Ref	871 (29.8%)	Ref
**Parity**				
Primiparous	296 (61.2%)	1.7	303 (39.8%)	1.76
Multiparous	1,186 (48.0%)	Ref	623 (27.3%)	Ref
**Type of delivery**				
Normal vaginal	1,396 (49.7%)	2.61	106 (53.7%)	2.91
Cesarean	165 (72.1%)	Ref	2008 (28.5%)	Ref
**Maternal age of delivery**	**Mean (±SD)**		**Mean (±SD)**	
	28.0^$^ (±6.4)	1*	28.4^@^ (±6.4)	0.99

* not significant since p value is ≥0.25.

$ no significant difference using t-test (p value: 0.87).

@ no significant difference using t-test (p value: 0.79).

The variables identified to be probable effect measure modifiers are parity and socio-economic status. Result of the stratified analysis for the association of pregnancy intention and breastfeeding initiation showed that socio-economic status has the potential to modify the odds ratio of mistimed pregnancy (p value: 0.005). However, parity is not a probable EMM on the odds ratio of both mistimed and unwanted pregnancy. On the other hand, results of the stratified analysis for the association of pregnancy intention showed that socio-economic status could also modify the odds ratio of unwanted pregnancy (p value: 0.042). Parity is not also a probable EMM.

The variables that passed the criteria for effect measure modification and confounding were then retained and fitted into the logistic regression model. In the model to determine the association of pregnancy intention and breastfeeding initiation, socio-economic status was assessed as effect measure modifier. The interaction term between unwanted pregnancy and socio-economic status exhibited a statistically significant likelihood ratio test with p value of 0.015. On the other hand, the interaction term between unwanted pregnancy and socio-economic status on breastfeeding duration exhibited a statistically significant likelihood ratio test with p value of 0.0486 (Table 2 and 3).

**Table 2:** The effect of different types of pregnancy intention on breastfeeding initiation stratified according to parity and socio-economic status

**Probable Effect Measure Modifiers**	**Mistimed Pregnancy**		**Unwanted Pregnancy**	
	**OR**	**85% CI**	**OR**	**85% CI**
**Parity**				
Primiparous	1.00	0.97-1.30	0.81	0.53-1.22
Multiparous	1.12	0.76-1.32	1.04	0.91-1.19
**Test for Homogeneity**	0.57		0.36	
**Socio-economic status**				
Low SES	1.37	1.15-1.64*	0.90	0.76-1.08
High SES	0.85	0.71-1.02	0.93	0 .77-1.12
**Test for Homogeneity**	0.005*		0.89	

*significant.

**Table 3:** The effect of different types of pregnancy intention to breastfeeding duration stratified according to parity and socio-economic status

**Probable Effect Measure Modifiers**	**Mistimed**		**Unwanted**	
	**OR**	**85% CI**	**OR**	**85% CI**
**Parity**				
Primiparous	1.01	0.78-1.08	0.66	0.42-1.03
Multiparous	0.92	0.77-1.33	0.82	0.70-0.95
**Test for Homogeneity**	0.64		0.49	
**Socio-economic status**				
Low SES	0.97	0.76-1.23	0.97	0.77-1.22
High SES	0.92	0.76-1.11	0.61	0.50-.74*
**Test for Homogeneity**	0.79		0.02*	

*significant.

Because socio-economic status has been identified as an effect measure modifier, changes in the stratum specific odds ratio estimates were used in order to properly assess confounding. In the model to determine the association between pregnancy intention and breastfeeding initiation, only parity was found to be a confounder since its exclusion from the model brought about a change in the stratum specific OR estimates that was greater than 10%. In the model to determine the association between pregnancy intention and breastfeeding duration, only parity and breastfeeding initiation were found as significant confounders (Table 4 and 5).

**Table 4:** Result of the assessment of confounding for the association of pregnancy intention and breastfeeding initiation among children belonging to households, by socio-economic status

**Confounders**		** Low SES**				** High SES**		
	**Mistimed**		**Unwanted**		**Mistimed**		**Unwanted**	
	**OR**	**% change**	**OR**	**% change**	**OR**	**% change**	**OR**	**% change**
Full model	**0.9020082**		**1.02067**		**0.9020082**		**1.02067**	
Parity	0.86	5%	0.9	12%	0.86	5%	0.9	12%
Education	0.9	0%	1.02	0%	0.9	0%	1.02	0%
Delivery type	0.86	5%	0.99	3%	0.86	5%	0.99	3%
Type of residence	0.86	5%	0.99	3%	0.86	5%	0.99	3%
Employment	0.86	5%	0.99	3%	0.86	5%	0.99	3%

**Table 5:** Result of the assessment of confounding for the association of pregnancy intention and breastfeeding initiation among children belonging to households with high socio-economic status

**Confounders**		** Low SES**				** High SES**		
	**Mistimed**		**Unwanted**		**Mistimed**		**Unwanted**	
	**OR**	**% change**	**OR**	**% change**	**OR**	**% change**	**OR**	**% change**
Full model	**1.444546**		**1.107328**		**0.9020082**		**1.02067**	
Parity	1.38	5%	1	10%	0.86	5%	0.9	12%
Education	1.45	0%	1.11	0%	0.9	0%	1.02	0%
Delivery type	1.44	0%	1.12	−1%	0.86	5%	0.99	3%
Type of residence	1.44	0%	1.12	−1%	0.86	5%	0.99	3%
Employment	1.44	0%	1.12	−1%	0.86	5%	0.99	3%

The final model for the association between pregnancy intention and breastfeeding initiation as shown in Table 6 indicates the effect of pregnancy intention on breastfeeding initiation differs by type of socio- economic status. Among children belonging to low SES, children born from mistimed pregnancies have higher odds of having late breastfeeding initiation (OR = 1.44; 90%CI: 1.17-1.78) compared to children born from wanted pregnancies. No significant effect of unwanted pregnancy was observed on breastfeeding initiation (1.12; CI: 0.91-1.38). Among children belonging to high SES, no significant effect of mistimed and unwanted pregnancies on breastfeeding initiation (OR = 0.85; CI: 0.69-1.06) (OR = 0.99; 90%CI: 0.80-1.23).

**Table 6:** Effect of Socio-economic status on the association of pregnancy intention and breastfeeding practices

**Pregnancy intention**	**Adjusted**	**odds ratio**
	**Adjusted odds Ratio**	
	**Low SES**	**High SES**
late breastfeeding initiation		
Wanted pregnancy	Reference	Reference
Mistimed pregnancy	1.44(CI:1.17-1.78)*	0.85 (CI: 0.69-1.06)
Unwanted pregnancy	1.12 (CI: 0.91-1.38)	0.99 (CI: 0.80-1.23)
Short breastfeeding duration		
Wanted pregnancy	Reference	Reference
Mistimed pregnancy	0.90 (CI:0.68-1.19)	0.83 (CI:0.67-1.04)
Unwanted pregnancy	1.01 (CI: 0.77-1.33)	0.60 (CI: 0.48-0.76)*

*significant.

Similarly, the final model for the association between pregnancy intention and breastfeeding duration as shown indicates the effect of pregnancy intention on breastfeeding duration also differs by socio- economic status. Among children belonging to high SES, the odds of having been breastfed for short duration is lower if they were born from unwanted pregnancies compared to children born from wanted pregnancies (OR = 0.60 CI: 0.48-0.76).

## Discussion

Tabulation of common socio-demographic characteristics and optimal breastfeeding practices suggests that mothers with higher levels of education, living in urban areas and are employed are more likely to initiate breastfeeding late and have shorter duration of breastfeeding. Urbanity which is related to higher level of education and employment drives mother to use convenient method of infant feeding like formula milk.

For the analysis of the independent association of pregnancy intention and breastfeeding initiation using regression analysis, it was found that children born from mistimed pregnancies are more likely to have late breastfeeding initiation compared to children born from wanted pregnancies (OR = 1.44; 90%CI: 1.17-1.78). However, this occurs only among children belonging to households with low socio-economic status. Among children belonging to households with high socio-economic status, no significant effect of pregnancy intention on breastfeeding initiation was observed.

The negative effect of mistimed pregnancies on prenatal care, immunization, infant feeding patterns and other desirable public health practices examined in many studies show that poor mothers with unplanned pregnancies experienced psychosocial pressures and stresses that inhibit them to perform public health practices like breastfeeding initiation[9].

On the other hand, children born from unwanted pregnancies are less likely to have short breastfeeding duration (OR = 0.60 90%CI: 0.48-0.76). However, this occurs only among children belonging to households with high socio- economic status. No significant effect of pregnancy intention on breastfeeding duration was observed among children belonging to households with low socio-economic status.

With regard to duration, children from high SES household, having been wanted during their mother’s pregnancy increases their chances of being breastfed for short duration. This finding negates the initial hypothesis of the study that children born from unwanted pregnancy are more likely to have short breastfeeding duration. Studies in the United States, Ghana and Australia have indicated that children born from mistimed and unwanted pregnancies are more likely to breastfeed for short duration [6-8]. However, there are several probable explanations to the observed deviation from findings of existing studies appraised.

First, the role of perception on breast milk may play an important factor. Mothers with wanted pregnancies are more likely to be excited and emotionally ready, thus, they are more likely to give the needs of the children which they perceive to be “best” or “sufficient”. Although majority of mothers are well informed on the benefits of breastfeeding, some believe it to be insufficient. Recent qualitative studies conducted in Mexico and Zambia indicated that significant number of mothers believe that breastfeeding alone is not sufficient as a method of feeding infants [12,13]. Thus, they resort to formula milk since they perceive it to be more sufficient than breast milk. The association of breastfeeding duration and pregnancy intention is not significant among children belonging to low SES households. This may be a reflection of the inability of low SES women to purchase infant formulas so that they are left with the most economical approach to feed their children – breastfeeding. Second, mothers with wanted pregnancies are more receptive to different sources of health and nutritional information. Therefore, these mothers have more choices on different forms of infant feeding. The themes and misleading claims in advertisements of infant formula milk may persuade these mothers to use formula milk instead [14]. Third, physicians play an important role on infant feeding practices of mothers. Mothers with wanted pregnancies are more likely to visit physicians for their prenatal and post natal care [6]. As such, their physicians may recommend infant formula milk which hinders them to use breast milk. Lastly, the occurrence of reverse causality is also probable. A mother whose last child is unwanted or mistimed would tend to practice lactational amenorrhea in order for her not to commit unwanted and mistimed pregnancy again in the near future. However, the current model tested did not address the possibility of reverse causality as this can be only achieved through prospective studies [15].

## Conclusion

The findings of this study suggests that among infants belonging to high SES, there is significant decrease in the odds of having short breastfeeding duration among children born from unwanted pregnancies compared to children born from wanted one. Thus, it is deemed necessary that reproductive health services should be further strengthened by increasing the availability and accessibility of effective family planning methods.

On the other hand, children belonging to low SES household, there is a significant increase in the odds of having late breastfeeding initiation among children born from mistimed pregnancies compared to children born from wanted pregnancies. Hence, it is very important to strengthen the promotion of the benefits of optimal breastfeeding practices among mothers. However, it is necessary that health education activities be localized or customized depending the target audience.

## Competing interest

The authors declare that they have no competing interests.

## Authors’ contribution

VGU have made substantial contribution in the design and conception of this paper. He drafted the manuscript, performed the data analysis and interpreted the results. On the other hand, MPB critically commented to improve the design, analysis and the direction of the paper. She served as the adviser of this research endeavor. Both authors read and approved the final manuscript.

## Pre-publication history

The pre-publication history for this paper can be accessed here:

http://www.biomedcentral.com/1471-2393/12/69/prepub
